# Roughage to Concentrate Ratio and *Saccharomyces cerevisiae* Inclusion Could Modulate Feed Digestion and In Vitro Ruminal Fermentation

**DOI:** 10.3390/vetsci7040151

**Published:** 2020-10-09

**Authors:** Kampanat Phesatcha, Burarat Phesatcha, Metha Wanapat, Anusorn Cherdthong

**Affiliations:** 1 Department of Animal Science, Faculty of Agriculture and Technology, Nakhon Phanom University, Nakhon Phanom 48000, Thailand; kampanatmon@gmail.com; 2 Department of Agricultural Technology and Environment, Faculty of Sciences and Liberal Arts, Rajamangala University of Technology Isan, Nakhon Ratchasima 30000, Thailand; Burarat_kat@hotmail.co.th; 3 Tropical Feed Resources Research and Development Center (TROFREC)Department of Animal Science, Faculty of Agriculture, Khon Kaen University, Khon Kaen 40002, Thailand; metha@kku.ac.th

**Keywords:** direct fed microbial, diet ratio, digestibility, ruminal fermentation, live yeast

## Abstract

The objective of this research was to investigate the effect of the roughage-to-concentrate (R:C) ratio and the addition of live yeast (LY) on ruminal fermentation characteristics and methane (CH_4_) production. The experimental design was randomly allocated according to a completely randomized design in a 4 × 4 factorial arrangement. The first factor was four rations of R:C at 80:20, 60:40, 40:60, and 20:80, and the second factor was an additional four doses of *Saccharomyces cerevisiae* (live yeast; LY) at 0, 2.0 × 10^6^, 4.0 × 10^6^, and 6.0 × 10^6^ colony-forming unit (cfu), respectively. For the in vitro method, during the incubation, the gas production was noted at 0, 1, 2, 4, 6, 8, 10, 12, 18, 24, 48, 72, and 96 h. The rumen solution mixture was collected at 0, 4, 8, 12, and 24 h of incubating after inoculation. Cumulative gas production at 96 h was highest in the R:C ratio, at 20:80, while the addition of LY improves the kinetics and accumulation of gas (*p* > 0.05). Maximum in vitro dry matter digestibility (IVDMD) and in vitro organic matter digestibility (IVOMD) at 24 h after incubation were achieved at the R:C ratio 20:80 and the addition of LY at 6 × 10^6^ cfu, which were greater than the control by 13.7% and 12.4%, respectively. Ruminal pH at 8 h after incubation decreased with an increased proportion of concentrates in the diet, whereas it was lowest when the R:C ratio was at 20:80. Increasing the proportion of a concentrate diet increased total volatile fatty acid (TVFA) and propionic acid (C3), whereas the acetic acid (C2) and C2-to-C3 ratios decreased (*p* < 0.05). TVFA and C3 increased with the addition of LY at 6 × 10^6^ cfu, which was greater than the control by 11.5% and 17.2%, respectively. No interaction effect was observed between the R:C ratio and LY on the CH_4_ concentration. The calculated ruminal CH_4_ production decreased with the increasing proportion of concentrates in the diet, particularly the R:C ratio at 20:80. The CH_4_ production for LY addition at 6 × 10^6^ cfu was lower than the control treatment by 17.2%. Moreover, the greatest populations of bacteria, protozoa, and fungi at 8 h after incubation were found with the addition of LY at 6 × 10^6^ cfu, which were higher than the control by 19.0%, 20.7%, and 40.4%, respectively. In conclusion, a high ratio of roughage and the concentrate and addition of LY at 6.0 × 10^6^ cfu of the total dietary substrate could improve rumen fermentation, improve feed digestibility, and reduce the CH_4_ production.

## 1. Introduction

In recent years, with increased consumer concern for health, quality animal products, and environmental effects, the European Union has prohibited antibiotics and synthetic chemicals as feed additives. Supplementation of direct fed microbial has been shown to improve animal performance due to its ability to modify feed utilization and the efficiency [1,2]. Live yeast (LY) is one of the most studied of those direct-fed microbes; specifically, *Saccharomyces cerevisiae* is used not only to improve production performance, but also to reduce the risk of transferring antibiotic resistance or possible human pathogens and to minimize the excretion of pollutants [3,4,5]. *S. cerevisiae* has been used for many years, to promote ruminal fermentation, minimize the loss of energy and nutrients, and thus increase the ruminant production system. Some researchers have found that supplementation of the *S. cerevisiae* enhances ruminant digestion of feed in a variety of ways, such as increasing nutrient digestibility [6], maximizing the ruminal volatile fatty acid (VFA) proportion, decreasing ammonia nitrogen (NH_3_-N) [7], alleviating pH fluctuation, and stimulating the population of ruminal microorganisms [8]. High portions of concentrate in the ration significantly decreased the ruminal pH and NH_3_-N concentration. The addition of yeast resulted in a numerical increased in the ruminal pH and concentration of VFA [9]. Kumprechtova et al. [10] mentioned that the daily inclusion of high-producing dairy cows with LY significantly increased milk production than the control group 3.6% (38.95 vs. 37.38 kg/day, respectively), without reducing the percentage of milk fat and protein. In early lactation, LY supplementation favorably affected the metabolic status and may have exerted a protective effect on the liver in high production. In addition, ruminal methanogenesis is a major metabolic pathway in the elimination of hydrogen. Enteric methane (CH_4_) is a waste of energy for the ruminant and a major source of greenhouse gas from livestock. The practical methods of minimizing its production are thus actively researched. LY has the potential to change the fermentation process in the rumen that reduces the formation of CH_4_ gas [9]. Chaucheyras et al. [11] reported a shift in H_2_ utilization from methanogenesis to reductive acetogenesis by yeast. *S. cerevisiae* strains have been selected for the specific reduction of ruminal CH_4_ production [11], whereas other strains may indirectly decrease CH_4_ production by improving ruminal fiber degradation and feed conversion efficiency [7]. An increased proportion of starch in the diet changes concentrations of ruminal VFAs so that less acetate and more propionate formed and there is limited supply of hydrogen for methanogenesis. As the proportion of propionic acid increases, the pH also decreases, which reduces methanogenic activity [12]. It was proposed that additions of LY with different roughage-to-concentrate (R:C) ratios, using treated rice straw with 2.0% urea + 2.0% lime ((Ca(OH)_2_) (ULTRS) as basal roughage, can improve rumen fermentation efficiency and reduce CH_4_ production. Therefore, the objective of the current study was to investigate the effect of the of LY addition and the R:C ratio, using ULTRS as basal roughage, on ruminal pH, ruminal fermentation, microbial population, and methane emission in in vitro gas production. 

## 2. Materials and Methods

The study design and plan strictly followed the norms of the Animal Ethics Committee of Nakhon Phanom University (permission No. AENPU A2/2560). This study primarily involved laboratory analysis of ruminant feeds, for which requisite permission was granted to collect the rumen fluid from animals based on the Thailand Ethics of Animal Experimentation of National Research Council.

### 2.1. Experimental Design and Dietary Treatments 

The experimental design was randomly allocated according to a completely randomized design (CRD) in a 4 × 4 factorial arrangement, with three replicates per treatment, including blank triplicates (medium only). Incubations were repeated on three separate days (runs). The first factor was four rations of R:C at 80:20, 60:40, 40:60, and 20:80, and the second factor was an additional four doses of *S. cerevisiae* (live yeast; LY), at 0, 2.0 × 10^6^, 4.0 × 10^6^, and 6.0 × 10^6^ colony-forming unit (cfu), respectively. The rice straw (*Oryza sativa* L.) (RS) was treated with 2.0% urea + 2.0% lime (ULTRS) (Thai Poly Chemicals Co., Ltd., Samut Sakhon, Thailand) by adding 2 g urea and 2 g lime in 100 mL of water to 100 g (91% DM) of RS as a roughage source. The amount of a urea–lime solution was sprayed onto a stack of whole straw bales, and then the stack was covered with a plastic sheet at least 10 days. As demonstrated in Table 1, the concentrate was formulated with ingredients. ULTRS and the concentrate diet were dried at 60 °C and passed through a screen (1 mm), to determine the chemical analysis of dry matter (DM), organic matter (OM), and crude protein (CP) by using standard methods [13]. The fiber content, especially neutral detergent fiber (NDF) and acid detergent fiber (ADF), was determined according to Van Soest et al. [14]. The yeast strain *S. cerevisiae* in this study was obtained from the That Phanom district, Nakhon Phanom Province, Thailand, as well as cultured and amplified by using a malt extract medium containing a 0.1 g chloramphenicol/L and 130 g malt extract. Then, the total viable yeast numbers were counted by using the spread plate method in the form of cfu according to Wang et al. [7]. The yeasts were then stored at 4 °C before the start of in vitro fermentation. 

### 2.2. Rumen and Substrate Inocula

The rumen fluid was collected from two Thai native beef cattle with an initial body weight of 200 ± 15 kg. Thai native beef cattle were also adapted to concentrate diet (14.1% CP and 78.8% total digestive nutrient, dry-matter basis) (at 0.5% of live weight), and RS treated with urea and lime was fed ad libitum. Two cattle are the same as mentioned before, were housed in separate pens, and received vitamin/mineral blocks and fresh water for 14 days consistently. On day 15, before morning feeding, 1000 mL of rumen fluid was obtained from each beef cattle, using a suction pump, and then pooled and strained into an Erlenmeyer flask from four layers of cheesecloth. An experimental feed sample was weighed into 50 mL bottles of each total mixed substrate (200 mg). Bottles with the mixtures of substrate treatment were received CO_2_ flushing and pre-warmed in a water bath at 39 °C for 96 h. Bottles were sealed with a rubber and aluminum cap and then incubated at 39 °C for 96 h. Ruminal fluid was combined with the artificial saliva solution in a proportion of 2:1 (mL/mL), at 39 °C, under continuous CO_2_ flushing, according to Makkar et al. [16]. A portion of the rumen-fluid medium (40 mL) was transferred into each bottle, and blanks with only rumen fluid were used; after that, the LY was added, respectively, and then incubated at 39 °C, in a water bath, as Blummen and Orskov [17] described.

### 2.3. In Vitro Gas Production and Fermentation Characteristics

The production of gas was measured immediately following incubation and after at 0, 1, 2, 4, 6, 8, 12, 24, 48, 72, and 96 h, as well as using a calibrated syringe and a pressure transducer. Cumulative-production-of-gas data were fitted to the model of Ørskov and McDonald [18], as follows:

y = a + b (1−e^−ct^)

where a = the gas production from the immediately soluble fraction, b = the gas production from the insoluble fraction, c = the gas production rate constant for the insoluble fraction (b), t = incubation time, (a + b) = the potential extent of gas production, and y = gas produced at time “t”. 

Fermented liquid was collected at 4 and 8 h post-incubation, to measure the pH, and then filtered through four layers of cheesecloth. Twenty-five milliliters of rumen fluid was separated into two portions; for a total direct count of bacteria, protozoa, and fungi, the first portion was fixed with a 10% formalin solution in a sterilized 0.9% saline solution. The populations of bacteria, protozoa, and fungi were illustrated by a haemacytometer [19]. The second portion was for NH_3_-N analysis and total volatile fatty acid (TVFA) using 2 mL of sulphuric acid (H_2_SO_4_) added to 18 mL of incubation medium centrifuged at 16,000× *g* for 15 min, and the supernatant was stored at −20 °C before NH_3_-N analysis (Kjeltech Auto 1030 Analyzer, Tecator, Sweden). VFA profiles (acetic acid (C2), propionic acid (C3), and butyric acid (C4)) were analyzed, using high-performance liquid chromatography (instruments by controller water model 600E, water model 484 UV detector, column Nova-Pak C18, column size 3.9 mm × 300 mm, mobile phase 10 mM H_2_PO_4_ (pH 2.5)), and the production of methane (CH_4_) was estimated by using the Moss et al. [20] method as follows: CH_4_ production = 0.45 (acetic acid) − 0.275 (propionic acid) + 0.4 (butyric acid). Equation indicates that the molar percentage of volatile fatty acids (VFAs) influences the production of methane in the rumen. Acetate and butyrate promote methane production, while propionate formation can be considered as a competitive pathway for hydrogen use in the rumen. The inocula after inoculation were filtered through pre-weighed Gooch crucibles, and residual dry matter was estimated. The percent loss in weight was determined and presented as in vitro dry matter degradability (IVDMD). The dried feed sample and residue left above was ashed at 550 °C, for determination of in vitro organic matter degradability (IVOMD), according to Tilley and Terry [21].

### 2.4. Statistical Analysis

All of the experimental data were analyzed as a 4 × 4 factorial arrangement, in a completely randomized design (CRD), and subjected to the general linear model (GLM) of SAS version 9.0 (SAS Inst. Inc., Cary, NC, USA) [22]. The statistical, model including R:C ratio, dose of *S. cerevisiae,* and interaction effects, was as follows: Yij = µ + Ai + Bj + ABij + εij, where Yijk is an observation, µ is the overall mean, A is R:C ratio effect (i = 1, 2, 3, 4), B is dose of *S. cerevisiae* effect (j = 1, 2, 3, 4), AB is the interaction effect of R:C ratio and dose of *S. cerevisiae*, and εij the residual effect. For all para meters, differences among treatments means were contrasted by Tukey’s multiple comparison test when *p* < 0.05 [23].

## 3. Results and Discussions

### 3.1. Chemical Composition of Experimental Feeds

Table 1 shows the chemical composition of experimental feeds. The mixture of concentrate was formulated by using soybean meal, cassava chip, and available local byproducts. The nutritive value of the concentrate was 87.8, 93.7, 14.1, 18.9, and 14.8% for DM, OM, CP, NDF, and ADF, respectively. RS is a byproduct of agriculture, which is abundant in the tropic, and can be used to feed ruminants, especially during the dry season because it lacks quality roughage. RS has low nutritional value, high fiber and lignin, low voluntary intake of feed, low digestibility, and low crude protein content, thus increasing the nutritive value of RS with urea, and straw treated with calcium hydroxide can be used to increase the production of ruminants [24]. Straw treatment with calcium hydroxide, along with a low urea level, was of particular interest. The price of calcium hydroxide (Ca(OH)_2_) or lime, an alkaline, is cheaper than urea, and it can provide enough nitrogen for microorganisms in the rumen. RS treated with 2% urea + 2% Ca(OH)_2_ in this study contains 5.8% CP, 71.4% NDF, and 51.3% ADF, similar to Polyorach et al. [25], who which reported that RS treated with urea and calcium hydroxide increased crude protein content, DM intake, the digestibility of nutrients and ruminal VFA concentration. There is potential for using urea calcium hydroxide treatments to enrich the nutritive value of RS.

### 3.2. In Vitro Gas Production Kinetics 

There was no interaction effect between R:C ratios and LY on the kinetics of gas (*p* > 0.05) (Table 2). Gas production kinetics, including gas production from the immediately soluble fraction (a), the insoluble fraction (b), the gas production rate constant for the insoluble fraction (c), and the potential extent of gas production (a + b) and accumulated gas production (96 h), were affected by the R:C ratio and LY addition (*p* < 0.05). Cumulative gas production at 96 h was highest in the R:C ratio, at 20:80. The inclusion of a high-concentrate proportion results in increases in the fermentation rate and extent [26]. Similarly, Kang et al. [27] found that cumulative gas production increased with the increasing proportion of concentrates in the diet. Moreover, the addition of LY improves the gas kinetics and accumulation of gas, and this could be due to the fact that LY might stimulate the rumen microbe and greater digestibility of the incubated substrate, thus resulting in improved gas production kinetics. Tang et al. [28] revealed that supplementation of a yeast culture increased the cumulative gas production. These results are in agreement with Wang et al. [7], who reported that supplementation of a yeast culture increased the total gas production when incubating different diet types. The inclusion of yeast may not only increase total gas production but also cause qualitative changes in the gases produced by increasing animals and rumen efficiency so as to contribute less negative environmental effects [29]. 

### 3.3. In Vitro Digestibility

The effect of the R:C ratio and LY addition on ruminal pH, NH_3_-N, and in vitro digestibility are shown in Table 3. There was no significant interaction effect between the R:C ratio and LY addition on in vitro digestibility. With an increasing concentrate proportion, the in vitro dry matter digestibility (IVDMD) and in vitro organic matter digestibility (IVOMD) increased (*p* < 0.05). These findings agreed with Kang et al. [27], in regard to improving in vitro digestibility by increasing the proportion of concentrates in the diet. This could be due to the resulting induction in microbial growth, which improved digestibility afterward. The degradability of IVDMD and IVOMD had also increased with the addition of the LY dose. Maximum IVDMD and IVOMD, at 24 h after incubation, were achieved at the R:C ratio 20:80 and the addition of LY at 6 × 10^6^ cfu, which were greater than the control by 13.7% and 12.4%, respectively. Similarly, Wang et al. [7] found that the addition of LY improved IVDMD and in vitro NDF disappearance (IVNDFD). Williams et al. [6] indicated that the inclusion of *S. cerevisiae* increased dry-matter digestibility when hay was fed to steers. Tang et al. [28] reported that the addition of *S. cerevisiae* with low-quality roughage enhanced in vitro digestibility. Moreover, the addition of *S. cerevisiae* at 2.5 g/d increased NDF and the digestibility of either extract in cattle [30]. Similarly, Cagle et al. [12] also observed that the addition of dry LY at 10 g/d in growing and finishing beef cattle increased DM and NDF digestibility. The fact that the digestibility of nutrients improved with the addition of *S. cerevisiae* could be due to the stimulation of the rumen microbe to a higher rate of feed digestion. Furthermore, it has been suggested that *S. cerevisiae* can scavenge the available oxygen on the surfaces of freshly ingested feeds to maintain metabolic activity, thereby reducing the potential for redox in the rumen [11,31,32].

### 3.4. Ruminal pH and Ammonia-Nitrogen (NH_3_-N) Concentration

The effect of the R:C ratio and LY addition on ruminal pH and the NH_3_-N concentration is shown in Table 4. There were no interactions between the R:C ratio and LY addition on ruminal pH and the NH_3_-N concentration in the present study. The pH value ranged from 6.2 to 6.9 for all treatments. However, the result showed that ruminal pH at 8 h after incubation decreased with an increased proportion of concentrates in the diet, whereas it was lowest when the R:C ratio was at 20:80. Similarly, Cherdthong et al. [33] revealed that the diets containing a high proportion of concentrate usually caused a marked reduction in ruminal pH, inhibited the digestion rate, and reduced the activity of cellulolytic bacteria. Ruminal pH is a major index representing the rumen environment’s internal homeostasis; hence, maintaining a consistently stable ruminal pH is essential to ensure optimal rumen ecology, rumen fermentation, and microbial growth. Ruminants typically have a highly developed system for maintaining a ruminal pH range of 6.0–7.0 [34]. Similarly, the present result demonstrated that, even though pH after 8 h incubation reduced, it maintained in the normal range for microbial activity. In general, pH stabilization is associated with decreased levels of lactic acid in the rumen. Stimulating lactic acid–utilizing bacteria could account for decreases in lactic acid concentrations induced by *S. cerevisiae* and the corresponding moderation of the rumen pH. The role of yeast is to stimulate lactate users, increase the number of lactate users, and serve as a competitor with lactate producers [8]. Moreover, it is interesting that the addition of LY increased ruminal pH (*p* < 0.05), and it is in agreement with a previous study by Dias et al. [35], who found that supplementing *S. cerevisiae* in the diet with high starch increased ruminal pH, whereas the concentration of lactate was reduced in lactating dairy cows. 

Bacteria and protozoa attack dietary protein in the rumen and rapidly degrade it into peptides, amino acids, and NH_3_-N. Ammonia is the principal source of nitrogen for microbial protein synthesis, and as the sole source of N, bacteria can grow with NH_3_-N. The current study found that the NH_3_-N concentration ranged from 17.1 to 24.1 mg/dL. The NH_3_-N was highest in the R:C ratio of 20:80. This could be due to the fact that a higher CP from high-concentrate diet ration may supply microbial breakdown into high concentrations of NH_3_-N than those with a low ratio concentrate diet [36]. Wanapat and Pimpa [37] suggested that an optimal concentration of NH_3_-N, ranging from 15 to 30 mg/dL, may enhance voluntary feed intake, microbial protein synthesis, digestibility of nutrients, and rumen ecology, whereas NH_3_-N deficiency inhibits the growth rate of bacteria. Furthermore, the addition of LY significantly reduced the NH_3_-N concentration (*p* < 0.05), which was similar to the results of Kumprechtova et al. [9], who found that the NH_3_-N concentration in the rumen was numerically lower in dairy cows supplemented with LY addition of *S. cerevisiae*. These data show that concentration of NH_3_-N may not be the microbial-growth-limiting factor. However, this result can be associated with higher total bacteria populations. The increase in VFA concentrations in this present study indicates enhanced microbial activity in terms of fermentation and agrees with previous findings regarding total bacteria populations [2,4]. The decrease in the ruminal NH_3_-N concentration seemed to be due to increased absorption of NH_3_-N into microbial proteins, probably because of the stimulation of microbial activity by *S. cerevisiae* [38,39].

### 3.5. Volatile Fatty Acid and Methane Production

Table 4 shows the TVFA, proportion of acetate, propionate, and butyrate. No interaction effect was observed between the R:C ratio and LY addition on TVFA and VFA profiles. The total concentrations of VFA ranged from 43.2 to 50.4 mM in all treatments and were similar to those observed by Wang et al. [7] and Cagle et al. [3]. Increasing the proportion of a concentrate diet increased TVFA and C3, whereas the C2 and the C2-to-C3 ratios decreased (*p* < 0.05). When the degradability of feed increased, the TVFA and proportion of C3 concentrations increased, whereas the proportion and ratio of C2 to C4 and C2 to C3 decreased, respectively [3,26,40]. This could be due to the fact that a concentrate contains a fraction of highly degradable carbohydrates, especially starch. A concentrate diet containing high starch tended to ferment toward C3, in which rumen bacteria fermented the soluble carbohydrate and starch in order to increase TVFA and C2, C3, and C4 in their cells [27].

In the current study, TVFA and C3 increased with the addition of LY at 6 × 10^6^ cfu, which was greater than the control by 11.5% and 17.2%, respectively. The C2 and C2-to-C3 ratios were lower than the control by 14.0% and 36.8%, respectively. Bakr et al. [41] found increasing TVFA and C3 concentrations in dairy cows fed with *S. cerevisiae.* The addition of *S. cerevisiae* also changed the molar proportion of VFA specifically the C3 concentration in the rumen, leading to an increase in the potential glucogenic for ruminants. In contrast, Mutsvangwa et al. [42] discovered the addition of *S. cerevisiae* on in vitro gas fermentation and indicated no change in the ratio of C2 to C3. This variation could be influenced by diverse yeast strains and the kind of diets used in different experiments.

The ability of LY in the rumen could assist in the growth of lactate-consuming and cellulolytic bacterial populations, sequentially aiding in the stabilization of the rumen and increasing the rumen’s capacity to digest fiber [43]. The addition of LY might have enhanced the rumen microbial population, resulting in a better fermentation of carbohydrates into VFAs [3].

No interaction effect was observed between the R:C ratio and LY on the CH_4_ concentration, as shown in Table 4. The calculated ruminal CH_4_ production decreased with the increasing proportion of concentrates in the diet, particularly the R:C ratio, at 20:80, which were in agreement with the report by Anantasook and Wanapat [26] and Kang et al. [27]. This could be due to the fact that a high proportion of C3 was caused by a decline in the production of CH_4_ and that the expected shift of H_2_ from the CH_4_ pathway made it available for use as C3 synthesis, which is nutritionally beneficial for ruminants [1].

In the current study, the CH_4_ production for LY addition at 6 × 10^6^ cfu was lower than the control treatment by 17.2%. Mutsvangwa et al. [42] stated that decreased CH_4_ production with yeast-containing rations may be due to increased C3 production that requires the use of metabolic hydrogen and thus reduces methanogenesis. LY has recently been suggested to minimize CH_4_, and the production of CH_4_ in the rumen has been shown to be decreased by inducing an acetogen to consume more hydrogen for the production of C2 [43,44]. These results agreed with the previous research of Lu et al. [45], who reported that the addition of LY reduced the production of CH_4_ in growing goats. In contrast, Wang et al. [7] found that adding LY to crop straw increased CH_4_ and that the elevation of CH_4_ production could result in increased fiber degradation under in vitro gas production. Elghandour et al. [46] conducted an in vitro experiment and proposed that variations in CH_4_ production between yeast cultures could be due to their varying protein, fat, fiber, and other material contents.

### 3.6. Rumen Microorganism

Table 5 shows the effect of ratio of R:C and LY addition on rumen microorganisms. There was no interaction between the R:C ratio and LY addition. Increasing the proportion of concentrates in the diet increased the total bacteria and protozoal population. This may be due to the fact that protozoal play a role in starch sequestration, decreasing the starch fermentation rate, and thereby maintaining the optimal ruminal pH for microbial growth. In addition, microbial bacteria in the rumen have been found to continually increase when a rapid fermentation carbohydrate is supplemented [27,35]. This is in agreement with Cherdthong et al. [33], who stated that the synthesis of ruminal microbial bacteria depends on an adequate supply of carbohydrate as an energy source and NH_3_-N for peptide bond synthesis. Anantasook and Wanapat [26] reported that high-level concentrate diet remarkably increased the rumen’s total bacteria. 

Moreover, the greatest populations of bacteria, protozoa, and fungi at 8 h after incubation were found with the addition of LY at 6 × 10^6^ cfu, which were higher than the control by 19.0%, 20.7%, and 40.4%, respectively. This shows that *S. cerevisiae* may provide growth factors, such as organic acid or vitamins, which might stimulate the cellulolytic bacterial population. Moreover, *S. cerevisiae* can improve rumen maturity and stabilize ruminal pH, which provides a better environment for the growth of rumen microbes, particularly bacteria and fungi-degrading cellulose [47]. Moreover, the addition of LY in dairy cows has increased the relative abundance of microorganisms used for cellulolytic, amylolytic, and lactate use [48,49].

## 4. Conclusions and Recommendations

In conclusion, a high ratio of concentrate and addition of LY at 6.0 × 10^6^ cfu of the total dietary substrate could improve in vitro gas production kinetics, nutrient digestibility, C3 concentration, and the microbial population, as well as reduce the CH_4_ production. The addition of LY improved rumen fermentation and reduced in CH_4_ production; hence, it has potential for antibiotics substitution and is beneficial to be used for feeding ruminant. However, further in vivo research should be conducted especially in lactating dairy cows and feedlot beef cattle. 

## Figures and Tables

**Table 1:** Ingredients and chemical composition of concentrate and 2% urea plus 2% calcium hydroxide (Ca(OH)_2_) treated rice straw (ULTRS) used in the experiment.

Item	Concentrate	ULTRS	Roughage: Concentrate Ratio			
			80:20	60:40	40:60	20:80
**Ingredient (kg of DM)**						
Cassava chip	65.0					
Rice bran	10.5					
Coconut meal	11.0					
Palm kernel meal	6.0					
Urea	3.0					
Molasses	2.0					
Mineral premix	1.0					
Salt	1.0					
Sulfur	0.5					
Chemical composition						
Dry matter (DM), %	87.8	50.6	86.4	85.6	85.1	84.7
% Dry matter						
Organic matter (OM)	93.7	90.1	92.9	92.2	91.6	91.0
Crude protein (CP)	14.1	5.8	12.5	10.9	9.2	7.6
Neutral detergent fiber (NDF)	18.9	71.4	29.6	40.1	49.7	60.3
Acid detergent fiber (ADF)	14.8	56.3	23.1	31.3	39.9	47.8
Total digestible nutrients (TDN) ^1^	78.9	-	-	-	-	-

^1^ Calculated value [15].

**Table 2:** The effect of roughage-to-concentrate (R:C) ratio with live yeast (LY) supplementation on gas kinetics and cumulative gas production.

Treatment	R:C ^1^	LY ^2^	Gas Kenetics ^3^				Gas (96 h) mL/0.2 g DM Substrate
			a	b	c	a + b	
1	80:20	0	−3.1	76.4	0.03	73.3	65.4
2		2	−2.7	74.3	0.03	66.1	64.3
3		4	−0.8	71.5	0.03	70.7	66.7
4		6	−2.2	73.4	0.03	71.7	66.1
5	60:40	0	−0.4	70.7	0.04	70.3	68.8
6		2	1.3	69.2	0.04	71.5	70.2
7		4	1.1	69.8	0.04	70.9	71.1
8		6	1.6	69.1	0.04	70.7	69.7
9	40:60	0	1.1	70.8	0.05	69.7	67.1
10		2	1.7	70.1	0.05	68.4	68.3
11		4	2.8	69.2	0.05	66.4	70.6
12		6	1.3	68.8	0.05	67.5	68.4
13	20:80	0	3.1	68.0	0.07	71.1	72.0
14		2	3.4	71.9	0.07	75.3	75.2
15		4	4.0	74.2	0.07	78.2	79.1
16		6	4.3	73.1	0.07	77.4	79.4
SEM			0.38	0.43	0.02	0.35	0.39
Comparison							
R:C			0.03	0.02	0.03	0.04	0.04
LY			0.02	0.0.01	0.02	0.04	0.03
Interaction			0.61	0.49	0.55	0.19	0.11

^1^ R:C, roughage-to-concentrate ratio; ^2^ LY, live yeast addition (×10^6^ colony-forming unit); ^3^ Gas kinetic, a, the gas production from the immediately soluble fraction; b, the gas production from the insoluble fraction; c, the gas production rate constant for the insoluble fraction (b); a + b, the gas potential extent of gas production; SEM, standard error of the mean.

**Table 3:** The effect of roughage-to-concentrate (R:C) ratio with live yeast (LY) supplementation on rumen ecology and in vitro digestibility.

Trt	R:C ^1^	LY ^2^	pH		NH_3_-N (mg/dL)	In Vitro Digestibility, %			
			4 h	8 h		IVDMD 12 h	IVDMD 24 h	IVOMD 12 h	IVOMD 24 h
1	80:20	0	6.72	6.52	19.1	61.0	64.6	71.6	77.4
2		2	6.86	6.61	18.6	63.6	67.4	72.8	78.8
3		4	6.85	6.76	17.0	65.6	69.6	73.3	79.3
4		6	6.88	6.81	17.2	64.2	68.0	73.0	79.0
5	60:40	0	6.54	6.49	20.5	63.2	67.0	68.8	74.4
6		2	6.53	6.50	19.4	64.4	68.2	70.3	76.1
7		4	6.65	6.69	18.6	65.6	69.6	71.6	77.4
8		6	6.62	6.71	18.5	65.4	69.4	71.2	77.0
9	40:60	0	6.41	6.15	22.9	67.6	71.6	75.1	81.1
10		2	6.45	6.30	22.3	76.3	80.9	78.2	84.6
11		4	6.49	6.56	21.4	77.5	82.1	78.8	85.2
12		6	6.50	6.58	20.9	76.5	81.1	78.3	84.7
13	20:80	0	6.42	6.20	24.1	70.9	78.1	79.2	80.6
14		2	6.43	6.22	24.0	72.3	86.7	82.2	88.8
15		4	6.49	6.25	22.8	73.0	88.4	83.4	90.2
16		6	6.49	6.28	21.6	72.7	88.8	84.2	91.0
SEM			0.38	0.57	0.77	1.97	2.43	1.85	2.47
Comparison									
R:C			0.02	0.02	0.04	0.009	0.02	0.03	0.009
LY			0.01	0.03	0.02	0.02	0.01	0.02	0.01
Interaction			0.14	0.61	0.80	0.39	0.45	0.82	0.78

^1^ R:C, roughage-to-concentrate ratio; ^2^ LY, live yeast addition (×10^6^ colony-forming unit); NH_3_-N, ammonia-nitrogen; pH4, pH at 4 h after incubation; pH 8, pH at 8 h after incubation; IVDMD 12 h, in vitro dry matter digestibility at 12 h after incubation; IVDMD24 h, in vitro dry matter digestibility at 24 h after incubation; IVOMD12 h, in vitro organic matter digestibility at 12 h after incubation; IVOMD 24 h, in vitro organic matter digestibility at 24 h after incubation; SEM, standard error of the mean.

**Table 4:** The effect of roughage-to-concentrate (R:C) ratio with live yeast (LY) supplementation on in vitro total volatile fatty acids (TVFA), VFA profiles, and methane (CH_4_) production.

Treatment	R:C ^1^	LY ^2^	Total VFA, (mM/l)	C2, (%)	C3, (%)	C4, (%)	C2:C3 Ratio	CH_4_ Production ^3^, mM
1	80:20	0	43.2	71.9	13.9	8.8	3.7	30.6
2		2	44.1	68.1	23.8	8.1	2.9	27.3
3		4	43.5	66.4	25.1	8.4	2.6	26.3
4		6	44.6	66.1	24.5	9.3	2.7	26.7
5	60:40	0	45.2	71.4	19.7	8.9	3.6	30.3
6		2	47.5	65.0	25.0	10.0	2.6	26.4
7		4	46.8	65.2	25.2	9.6	2.6	26.3
8		6	47.1	64.9	26.6	8.5	2.4	25.3
9	40:60	0	43.9	70.1	22.5	7.4	3.1	28.3
10		2	45.8	65.0	26.8	8.2	2.4	25.2
11		4	46.3	65.8	26.4	7.8	2.5	25.5
12		6	47.1	61.3	27.1	11.6	2.3	24.8
13	20:80	0	45.2	67.8	26.1	6.2	2.6	25.8
14		2	48.1	62.9	29.7	7.4	2.1	23.1
15		4	48.2	59.1	30.4	10.5	1.9	22.4
16		6	50.4	59.0	30.6	10.4	1.9	22.0
SEM			2.04	0.75	0.04	0.32	0.08	0.13
R:C			0.009	0.009	0.009	0.02	0.008	0.007
LY			0.04	0.03	0.03	0.76	0.04	0.04
Interaction			0.52	0.19	0.31	0.55	0.34	0.37

^1^ R:C, roughage-to-concentrate ratio; ^2^ LY, live yeast addition (×10^6^ colony-forming unit); C2, acetic acid; C3, propionic acid; C4, butyric acid; ^3^ calculated according to Moss et al. [20], CH_4_ production = 0.45 (acetic acid) − 0.275 (propionic acid) + 0.4 (butyric acid); SEM, standard error of the mean.

**Table 5:** The effect of roughage-to-concentrate (R:C) ratio with live yeast (LY) supplementation on microbial population.

Treatment	R:C ^1^	LY ^2^	Bacteria (×10^10^ Cells/mL)		Protozoa (×10^5^ Cells/mL)		Fungi (×10^3^ Cells/mL)	
			4 h	8 h	4 h	8 h	4 h	8 h
1	80:20	0	9.5	8.8	4.0	4.2	1.3	1.4
2		2	10.3	11.0	4.6	5.3	1.9	1.9
3		4	10.9	11.3	4.5	5.7	1.8	2.1
4		6	10.8	12.1	4.6	5.8	1.9	2.1
5	60:40	0	10.3	13.4	4.4	5.6	1.7	1.8
6		2	12.1	14.7	5.3	5.9	2.0	2.2
7		4	14.4	17.4	6.3	6.5	2.6	2.9
8		6	15.3	18.8	6.1	6.5	2.7	2.8
9	40:60	0	13.7	16.1	6.0	7.0	2.3	2.6
10		2	15.1	17.8	6.9	7.1	2.6	4.0
11		4	16.5	19.8	7.8	7.9	2.5	3.8
12		6	16.9	20.9	7.7	8.0	2.8	4.2
13	20:80	0	17.7	19.5	7.0	8.2	2.1	4.7
14		2	18.3	20.7	7.3	8.5	2.3	5.1
15		4	19.2	22.1	8.6	9.0	2.6	5.6
16		6	20.3	23.2	8.8	9.9	2.9	6.6
SEM			0.95	0.87	0.28	0.53	0.62	0.70
Comparison								
RC			0.009	0.008	0.008	0.008	0.03	0.02
LY			0.008	0.008	0.009	0.009	0.18	0.32
Interaction			0.11	0.14	0.39	0.65	0.29	0.44

^1^ R:C, roughage: concentrate ratio; ^2^ LY, live yeast addition (×10^6^ colony-forming unit); SEM, standard error of the mean.
